# Biodegradable FePS_3_ nanoplatform for efficient treatment of osteosarcoma by combination of gene and NIR-II photothermal therapy

**DOI:** 10.1186/s12951-023-01961-9

**Published:** 2023-07-13

**Authors:** Tingting Luo, Mingyang Jiang, Ziqiang Cheng, Yuntao Lin, Yuling Chen, Zhenyu Zhang, Jian Zhou, Wenhua Zhou, Xue-Feng Yu, Shuchun Li, Shengyong Geng, Hongyu Yang

**Affiliations:** 1 Guangdong Provincial High-level Clinical Key Specialty, Guangdong Province Engineering Research Center of Oral Disease Diagnosis and Treatment, The Institute of Stomatology, Peking University Shenzhen Hospital, Shenzhen Peking University-The Hong Kong University of Science and Technology Medical Center, Shenzhen, Guangdong China; 2 Materials and Interfaces Center, Shenzhen Institutes of Advanced Technology, Chinese Academy of Sciences, Shenzhen, 518055 China; 3 Department of Applied Physics, School of Science, East China Jiaotong University, Nanchang, 330013 China

**Keywords:** FePS_3_ nanoplatform, NIR-II photothermal therapy, Gene therapy, Combination therapy, Osteosarcoma

## Abstract

As a common tumor with high incidence, osteosarcoma possesses extremely poor prognosis and high mortality. Improving the survival of osteosarcoma patients is still a great challenge due to the precipice of advancement in treatment. In this study, a combination strategy of gene therapy and photothermal therapy (PTT) is developed for efficient treatment of osteosarcoma. Two-dimensional (2D) FePS_3_ nanosheets are synthesized and functionalized by poly-L-lysine-PEG-folic acid (PPF) to fabricate a multifunctional nanoplatform (FePS@PPF) for further loading microRNAs inhibitor, miR-19a inhibitor (anti-miR-19a). The photothermal conversion efficiency of FePS@PPF is up to 47.1% under irradiation by 1064 nm laser. In vitro study shows that anti-miR-19a can be efficiently internalized into osteosarcoma cells through the protection and delivery of FePS@PPF nanaocarrier, which induces up-regulation of PTEN protein and down-regulation p-AKT protein. After intravenous injection, the FePS@PPF nanoplatform specifically accumulates to tumor site of osteosarcoma-bearing mice. The in vitro and in vivo investigations reveal that the combined PTT-gene therapy displays most significant tumor ablation compared with monotherapy. More importantly, the good biodegradability promotes FePS@PPF to be cleared from body avoiding potential toxicity of long-term retention. Our work not only develops a combined strategy of NIR-II PTT and gene therapy mediated by anti-miR-19a/FePS@PPF but also provides insights into the design and applications of other nanotherapeutic platforms.

**Supplementary Information:**

The online version contains supplementary material available at 10.1186/s12951-023-01961-9.

## Introduction

Osteosarcoma, most commonly occurs in adolescents and adolescents, is a primary malignant bone tumor [1]. Traditional medical treatments including surgery and chemotherapy result in a five year survival rate of only about 60% [2]. Moreover, adjuvant chemotherapy used to kill residual tumor cells after surgery also harm normal cells and may cause drug resistance, resulting in recurrence and endless suffering to patients [3–5]. Despite the novel therapeutic strategies that have been constantly investigated, there is limited progress for improving the prognosis in osteosarcoma patients [6]. Thus, it remains clinically urgent to explore effective strategies for osteosarcoma.

MicroRNAs (miRNAs), a length of 18–25 nucleotides, are endogenous non-coding RNA and participate in basic biological processes such as cell proliferation and differentiation through regulating gene expression.[7, 8] Many studies have fully verified that miRNAs can regulate cell proliferation of osteosarcoma [9, 10]. Thus, anti-sense miRNA and miRNA mimetics have capability to serve as gene therapy for osteosarcoma. However, the inherent obstacles associated with small RNAs, for example poor intracellular uptake and rapid blood clearance, severely limit their in vivo applications [11–14]. An effective nanocarrier with good biosafety is urgently needed to target transport the small RNAs to tumor site preventing their enzymatic degradation upon intravenous administration.

Nanoplatforms based on two-dimensional (2D) nanomaterials can be developed to overcome these challenges considering the large specific surface area to load and deliver small RNAs [15–19]. More importantly, the integration of photo-responsive 2D nanomaterials mediated photothermal therapy (PTT) and small RNAs mediated gene therapy can achieve ideal treatment of osteosarcoma. For example, gold nanorods-based GNR@siRNA/CPP, MoS_2_-PEG-PEI and WS_2_@PEI nanoplatforms are developed to treat tumors including osteosarcoma by the combination of PTT and gene therapy [20–22]. However, the non-biodegradability of this kind of nanomaterials induces them cannot be excreted naturally, which brings potential in vivo toxicities. As a new family of 2D nanomaterials, metal phosphorus chalcogenides (MPX_3_) such as FePS_3_ and FePSe_3_ nanosheets which have a wide bandgap and exhibit a strongly absorption in the second window of near-infrared (NIR-II, 1000–1350 nm) light have been recently reported to serve as PTT agents [23, 24]. In consideration of deeper penetration depth and higher maximal permissible exposure (MPE), NIR-II light is more suitable for clinical application compared to NIR-I (650–950 nm) light [25, 26]. Therefore, the construction of a multifunctional nanoplatform based on biodegradable 2D MPX_3_ may provide a potentially powerful treatment against osteosarcoma by the combination of gene therapy and PTT.

**Scheme 1:**
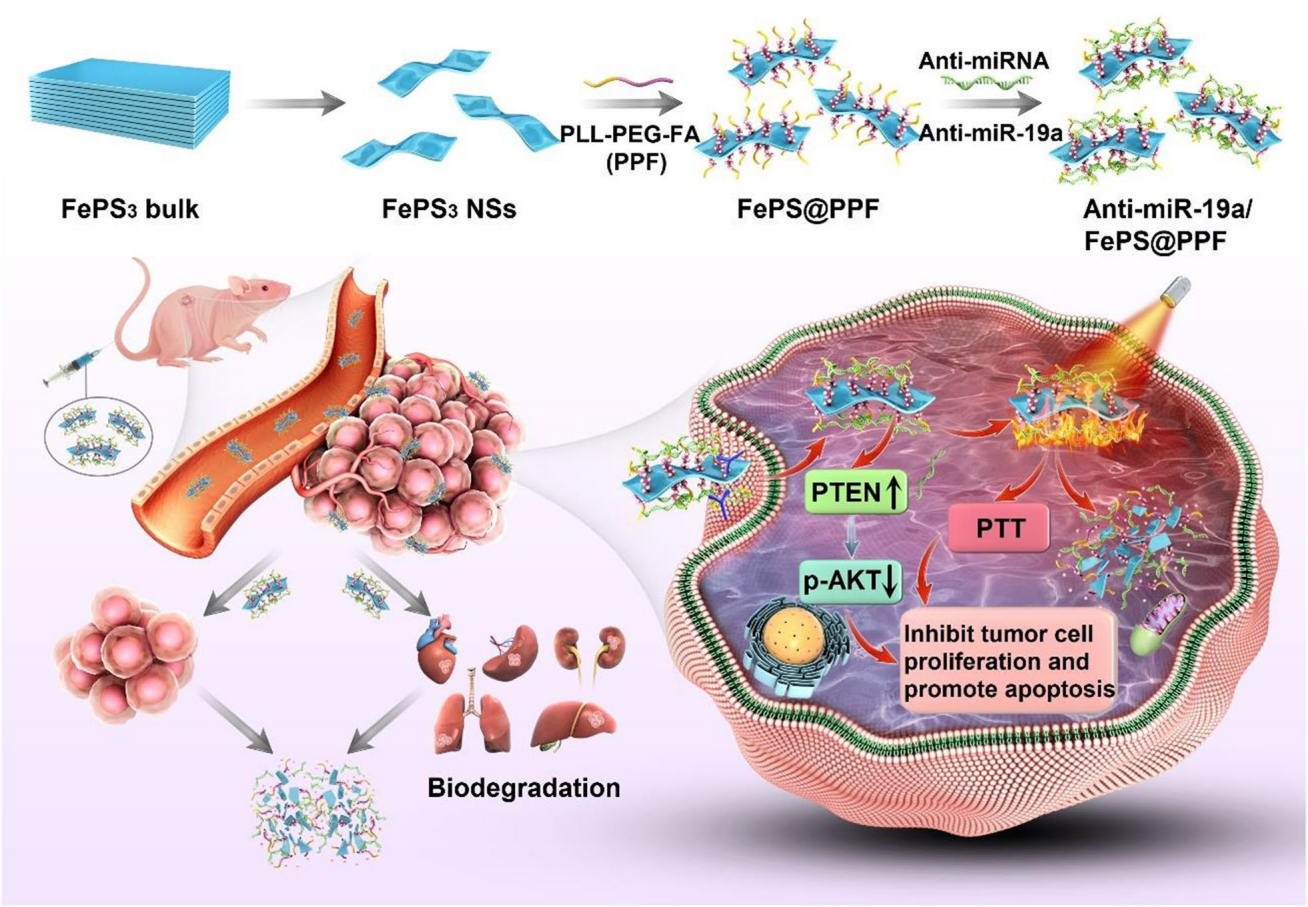
The fabrication of anti-miRNA loaded FePS_3_ nanoplatform (anti-miR-19a/FePS@PPF) for combination treatment of osteosarcoma

In this study, FePS_3_ nanosheets (FePS NSs) are prepared and functionalized by a PEG-conjugated poly-L-lysine-folic acid (FePS@PPF) to fabricate a novel tumor targeting miRNA delivery system (Scheme 1). Under irradiation of 1064 nm laser, FePS@PPF exhibits high photothermal conversion efficiency of 41.7%. Recently, researchers have indicated that miR-19a is significantly upregulated in human osteosarcoma and plays a critical role in osteosarcoma evolution [27–29]. After loading miR-19a inhibitor (anti-miR-19a), both the in vitro and in vivo experiments reveal synergetic effects of gene-photothermal therapy with good biosafety induced by anti-miRNA/FePS@PPF. The mechanisms regarding the killing of osteosarcoma cells are also investigated.

## Materials and methods

### Materials

Iron, red phosphorus, sulfur and iodine powders (99.99%), and NMP (N-Methyl-2-pyrrolidone) were bought from Aladdin Reagents. PLL-PEG, PLL-PEG-FA were purchased from Ruixi Co., Ltd (Xi’an, China). Anti-miR-NC, the negative control of microRNAs inhibitor, miR-19a inhibitor (anti-miR-19a), and Cy5.5-labeled anti-miR-19a were from RiboBio Co., Ltd (Guangzhou, China). FITC and Cy5.5 fluorescence dyes were obtained from Lumiprobe (Maryland, USA). The cell culture reagents including DMEM mediem and fetal bovine serum (FBS) and so on were from Gibco (AG, Switzerland). Beyotime (Shanghai, China) provided Calcein-AM, PI and DAPI staining solutions, and Cell Counting Kit-8 (CCK-8). The antibodies for immunostaining were from Cell Signaling Technology (Maryland, USA). Other chemical reagents at analytical reagent grade were directly used.

### Synthesis of FePS_3_ crystals and nanosheets

The FePS_3_ crystals were prepared using chemical vapor transport (CVT) technique. High-purity iron, red phosphorus and sulfur powders with the molar ratio of 1:1:3 (around 1.37 g in total), and the transport agent (iodine, 20 mg) were filled together in a quartz ampule with dimeter of 18 mm, length of 100 mm, wall thickness of 2 mm followed by seal under high vacuum (below 5 × 10^− 4^ Torr). Subsequently, the sealed quartz ampule was placed in a two-zone furnace and heated to 700 °C for 5 days. Finally, the two-zone furnace was cooled to room temperature in 8 h, and the bulk FePS_3_ crystals were obtained.

The FePS NSs were synthesized from bulk FePS_3_ crystals by a liquid exfoliation strategy. Briefly, 100 mg of FePS_3_ crystals were fully ground and then exfoliated by probe sonication in 100 mL of NMP for 12 h in a bath at 6 °C. After sonication, the precipitate between 9000 and 14,000 rpm was collected to obtain FePS NSs. Before using, washing with ethanol and water three times each was carried out.

### Functionalization of FePS NSs

1 mg of PLL-PEG or PLL-PEG-FA was mixed with 200 µg of FePS NSs dispersed in 5 mL water, sonicated for 30 min followed by stirring for 3 h. The obtained FePS@PP and FePS@PPF were washed to remove the excess PLL-PEG and PLL-PEG-FA. Afterwards, 0.6 nmol of anti-miR-NC or anti-miR-19a was added to 100 µg of FePS@PPF in 2 mL water, and magnetically stirred for 4 h at room temperature. Ultimately, functionalized anti-miRNA/FePS@PPF was collected after washing and centrifugating.

To synthesize FITC-labeled FePS@PPF, 0.1 mg of FePS@PPF and 1.0 mg of FITC dye were dispersed in 10 mL of water, magnetically stirred for 4 h. Then the mixture was washed with water to remove unreacted FITC.

### Characterizations

JEM-3200FS (JEOL, Japan) was used to take the transmission electron microscopy (TEM) images at 200 kV. Size distribution and zeta potential were determined using Zetasizer 3000 HAS (Malvern Ltd., UK). Atomic force microscopy (AFM) was performed on Bruker Multimode 8 with the drop-cast flakes on a Si/SiO_2_ substrate. XRD (X-ray diffraction) analysis was performed by the SmartLab X-ray diffractometer (Rigaku, Japan). The UV-Vis-NIR absorption were carried out by U-3900 spectrophotometer (Hitachi, Japan). The concentration of FePS NSs was measured with ICP-OES (7000DV, PerkinElmer, USA). Fourier Transform infrared spectroscopy (FT-IR) spectra were detected by MDTC-EQ-M13-01 (Thermo, USA).

### Photothermal effects

The FePS@PPF dispersed in water (0, 15, 25, 50 µg/mL) were exposed to 1064 nm laser for 10 min with a power density of 1.0 W/cm^2^. The temperature was monitored using the Ti27 infrared thermal imager (Fluke, USA). Additionally, 50 µg/mL of FePS@PPF solution was irradiated at 0.5, 1.0, and 1.5 W/cm^2^. The temperature changes during the rise and natural cooling processes were recorded.

### Photothermal conversion efficiency of FePS@PPF

The photothermal conversion efficiency (*η*) can be calculated by Eqs. 1–4.

1 $$\eta {\text{ = (hS(Tmax}}\, - \,{\text{Tsurr)}}\, - \,{\text{Qdis)/I(1}}\, - \,{\text{10}}\, {^{-\text{A}}})$$

2 $$hS = {\text{ }}\sum mC_{{\text{p}}} /\tau _{{\text{S}}}$$

3 $$\tau S{\text{ }} = {\text{ }} - {\text{ }}t/ln\theta$$

4 $$\theta = {\text{ }}\left( {T - T_{{{\text{surr}}}} } \right)/\left( {T_{{{\text{max}}}} - T_{{{\text{surr}}}} } \right)$$

where *h* is heat transfer coefficient, *S* is the area of container, *τ*_S_ is the time constant of system heat transfer, *m* is mass of 1 g, *C*_p_ (4.2 Jg^–1^ °C^–1^) is specific heat capacity of water, and *τ*_S_ = 263.77 s is obtained from Fig. 2f. *hS* is obtained from Eq. 2 (*hS* = 1*4.2/263.77 = 15.92 mW/°C), *Q*_dis_ is measured independently to be 74.84 mW, *T*_max_ is the equilibrium temperature of FePS@PPF and *T*_surr_ is the ambient temperature. *I* is 1.0 W/cm^2^ and *A* refers to absorbance of FePS@PPF at 1064 nm (*A*_1064_ = 0.568). Accordingly, *η* = {[15.92*(49.0-27.4)-74.84]/[1000*(1–10^− 0.568^)]}*100%= 47.1%.

### Intracellular uptake

5 × 10^4^ cells per dish of the HOS cells were seeded and cultured in confocal dishes overnight. After incubation with 25 µg/mL of FITC-labeled FePS@PPF or Cy5.5-labeled anti-miR-19a/FePS@PPF for 6 h, washing with PBS was carried out and fixing the cells with 4% PFA. The nuclei were stained by DAPI. The images were taken using confocal microscope (Leica stellaris 5, GER).

### In vitro antitumor efficiency

HOS and MG63 cells were seeded with 1 × 10^4^ cells per well in 96-well plates and cultured overnight. Different concentrations of FePS@PPFs (0, 6, 12, 25 and 50 µg/mL) were added and treated cells for 48 h. Then, a CCK-8 assay was used to determine cell viabilities. For antitumor assay, the medium containing 25 µg/mL (FePS@PPF concentration) of anti-miR-NC/FePS@PPF or anti-miR-19a/FePS@PPF was used to treat cells for 6 h. Then, the samples were removed by adding fresh medium, and exposed to 1064 nm laser for 10 min, 1.0 W/cm^2^. Incubation for another 48 h was performed, and CCK-8 assay was conducted to analyze cell viability. In addition, cells were treated as described above and co-stained with Calcein-AM/PI (5 µg/mL) at 37 ^o^C for 10 min. Subsequently, fluorescent images indicating live/dead cells were taken by IX71 (Olympus, Japan). The cell apoptosis after different treatments was measured by flow cytometry (BD FACSCelesta) using the Annexin V-FITC Apoptosis Kit.

### Western blot

Cells were lysed and the protein was extracted with radio immunoprecipitation lysis buffer on ice. BCA assay was used to determine the protein concentrations and Western blot was conducted using 12% SDS-PAGE. Bovine serum albumin was used to block the polyvinylidene fluoride membranes (0.45 mm) and then the membranes were incubated with different primary antibodies overnight at 4 °C. After washing, the primary antibodies-coated membranes were conjugated with secondary antibody at room temperature for 1 h. Finally, membranes were detected by chemiluminescence imaging (Bio-Rad, Singapore) and the bands were normalized to beta-actin level.

### Animals and construction of xenograft tumor models

Balb/c nude mice, which were female and about 4–6 weeks old were purchased from a company of Laboratory Animal Technology (Charles River Co., Ltd., China) and raised in SPF laboratory. The animal experiments were approved by Administrative Committee of SIAT (Shenzhen Institutes of Advanced Technology, Chinese Academy of Sciences) responsible for supervising of animal research.

HOS cells (1 × 10^8^/mL) were dispersed in PBS and the cell suspension (100 µL) was seeded into the right back side of mice. The formula: volume (V) = length × width^2^/2 was used to calculate the volume of tumor.

### In vivo biodistribution and photothermal effects

Cy5.5-labeled FePS@PP or FePS@PPF was intravenously injected with 10 mg/kg FePS NSs for tumor-bearing mouse. The in vivo fluorescence images were acquired by Caliper IVIS Spectrum (PerkinElmer, USA) at designed time point (3, 6, 12, 24, 48 h). The ex vivo fluorescence of heart, liver, spleen, lung, kidney and tumor was detected at 24 h post-administration. All images were analyzed and calculated by Living Image software.

Mice were anaesthetized after 24 h-injection of PBS, FePS@PP or FePS@PPF, and the tumor sites were irradiated by 1064 nm laser for 5 min, 1.0 W/cm^2^. The temperature of tumor site was monitored using an infrared thermal imager.

### In vivo synergistic anti-tumor effects

When the tumor models were established, the mice were divided randomly into six groups with 5 mice in each group: (1) PBS (control), (2) anti-miR-NC/FePS@PPF, (3) anti-miR-19a/FePS@PPF, (4) PBS + NIR, (5) anti-miR-NC/FePS@PPF + NIR, (6) anti-miR-19a/FePS@PPF + NIR. On day 0, 100 µL of PBS, anti-miR-NC/FePS@PPF or anti-miR-19a/FePS@PPF was injected *via* the tail vein at the concentration of 10 mg/kg FePS NSs once a week. On day 1 and day 7, mice in group (4), (5) and (6) were anaesthetized and exposed to 1064 nm laser (1.0 W/cm^2^, 5 min). The tumor length, width and body weight were measured every 2 days. On day 14, mice were sacrificed, tumor as well as main organs (heart, liver, spleen, lung, kidney) were collected and fixed in 4% PFA for histopathological and immunohistochemical analyses.

### In vivo clearance and biosafety evaluation

At 0-, 1-, 3-, 7- and 14-days after intravenous injection of FePS@PPF (10 mg/kg), the main organs were removed, digested in HNO_3_ and analyzed by ICP-OES. The concentrations of elements Fe, P and S in the main organs were determined to assess the biodegradability of FePS@PPF.

On day 14, 0.6 mL of blood sample was collected from venous plexus of eye socket into heparinized tubes. The serum samples were obtained by centrifugation at 3000 rpm, 15 min, 4 ℃. The blood biochemistry assay which is related with liver and renal function was detected at Wuhan Servicebio Technology Co., Ltd. The H&E staining of sections were performed to observe the tissue morphology.

### Statistical analysis

All quantitative results were presented as mean ± SD from three independent experiments. Statistical comparisons were analyzed by SPSS software (Chicago, USA) by Tukey’s post-test and one-way ANOVA analysis. *p < 0.05, **p < 0.01.

## Results and discussion

### Characterizations

**Fig. 1:**
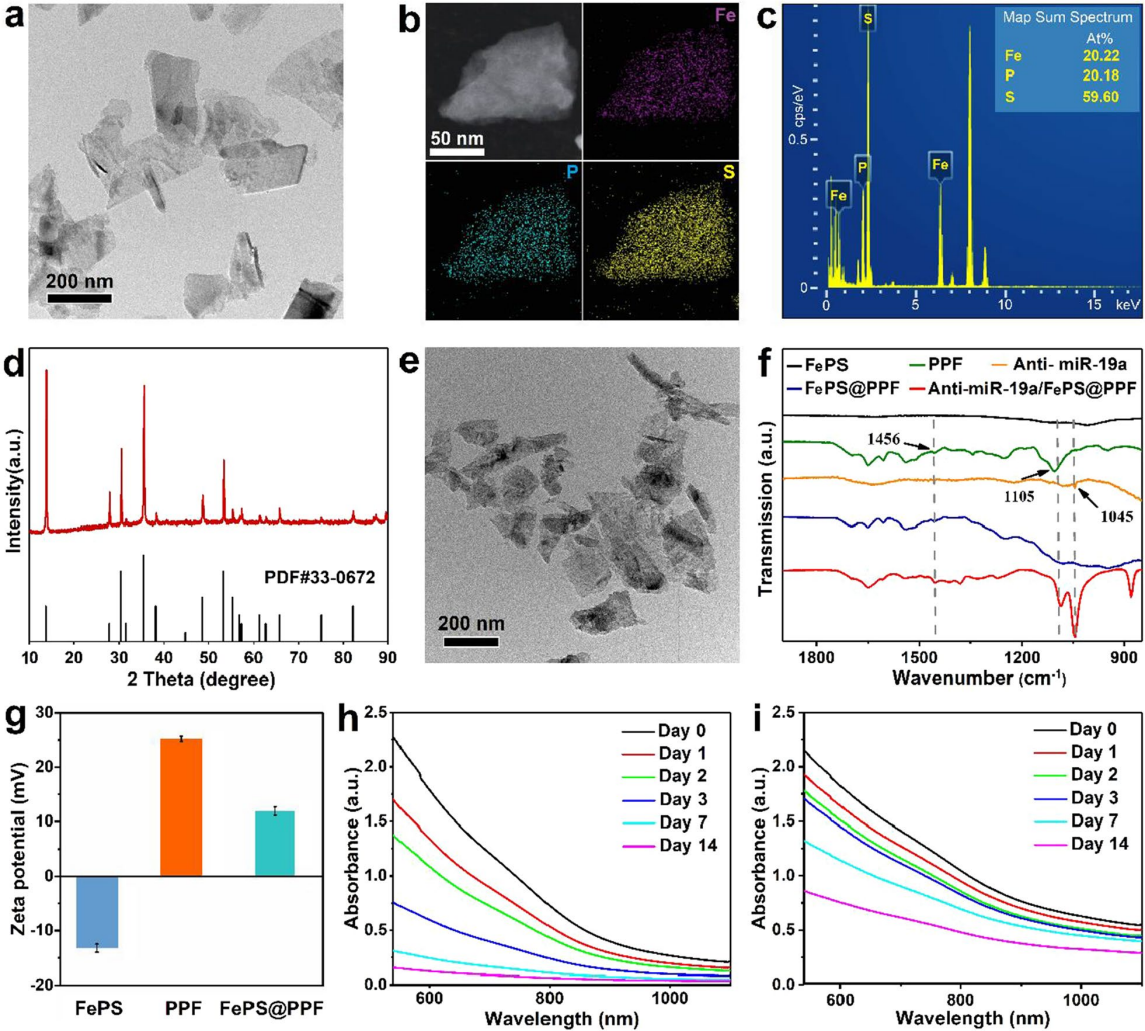
Characterization of nanomaterials. **a** TEM image of FePS NSs. **b** Elemental mapping obtained from TEM. **c** Map sum sepctrum of FePS NSs. **d** XRD patterns of the synthesized FePS NSs. **e** TEM image of FePS@PPF. **f** FT-IR spectra of bare FePS NSs, PPF, anti-miR19a, FePS@PPF and anti-miR-19a/FePS@PPF. **g** Zeta potentials of FePS NSs, PPF and FePS@PPF (n = 3). **h** Absorption spectra of FePS NSs and **i** FePS@PPF after storage in PBS for 14 days

The TEM image shows good dispersion of FePS NSs with a unique 2D structure and a size of about 200 nm (Fig. 1a). The average diameter is 206.1 nm with PdI of 0.16 by dynamic light scattering (DLS) analysis (Additional file 1: Fig. S1a) and the thickness is measured to be 13.8 nm by AFM (Additional file 1: Fig. S1b). The elemental mapping confirms the composition of Fe, P, and S (Fig. 1b), and the map sum sepctrum verifies that the ratio of Fe, P, S in FePS NSs is 1:1:3 (Fig. 1c). XRD analysis indicated that the result of FePS NSs is coordinated with the standard pattern (PDF card No.33–0672) (Fig. 1d). All these results indicate the successful synthesis of FePS NSs.

To enhance tumor targeting and prolong in vivo circulation lifetime, the surface of FePS NSs is modified by poly(l-lysine)-poly(ethylene glycol)-folate (named as FePS@PPF). The FePS@PPF maintains a unique 2D structure as that of bare FePS NSs with good dispersion and similar size (Fig. 1e). As revealed by FT-IR, FePS@PPF exhibits characteristic peaks at 1105 cm^− 1^ and 1456 cm^− 1^ which are consistent with those of pure PPF (Fig. 1f) compared to bare FePS NSs, comfirming the surface conjugation of PPF on FePS NSs (Fig. 1f). The zeta potential of FePS@PPF changes from − 13.2 to + 11.9 mV after the coating of PPF due to the electrostatic adsorption between FePS NSs and PPF (Fig. 1g). The anti-miR-19a/FePS@PPF exhibit characteristic peaks consistent with pure anti-miR-19a at 1045 cm^− 1^, suggesting successful loading of anti-miR-19a (Fig. 1f). In addition, the stability of FePS NSs and FePS@PPF dispersed in PBS is also evaluated. The absorbance of FePS NSs is reduced significantly and the color of solution obviously becomes lighter over time (Fig. 1h). In contrast, the FePS@PPF solution exhibits significantly improved stability with slight change in the absorbance (Fig. 1i and Additional file 1: Fig. S2). The hydrophilic polymer PLL-PEG-FA on the one hand endows FePS@PPF with good dispersion in PBS, and on the other hand the presence of PEG shell on the surface can decrease the degradation rate of FePS@PPF. In fact, PEG modification is a classical approach to improve the stability of nanomaterials in a physiological environment [30]. The steric hindrance of the hydrophilic PEG makes the degradation of PEG-functionalized nanomaterials start from their interior, whereas the materials without surface modification degrade from their external surface [31].

.

### NIR-II photothermal properties of FePS@PPF

**Fig. 2:**
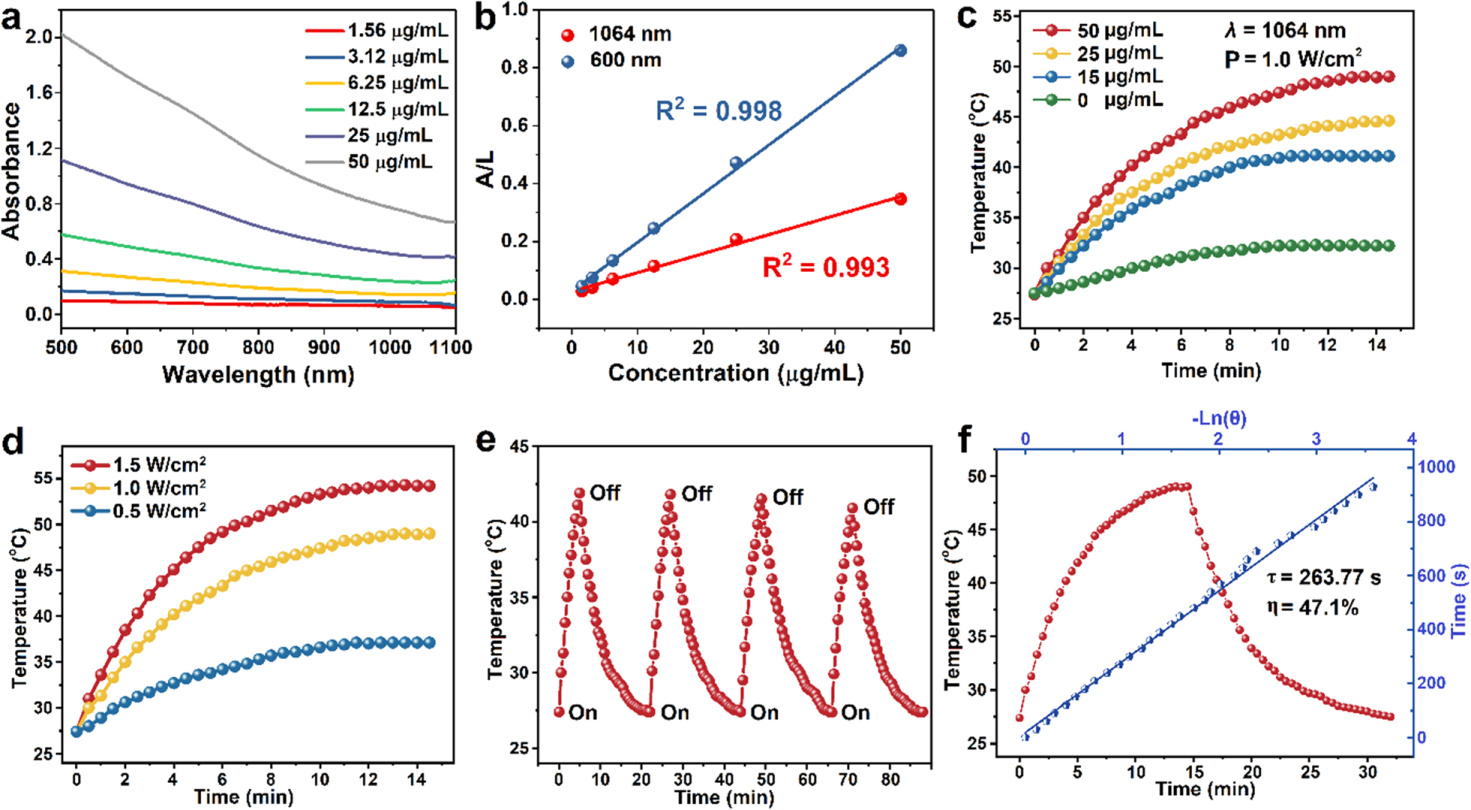
NIR-II photothermal effects of FePS@PPF. **a **Absorption spectra at different concentrations. **b** Normalized absorbance at 1064 nm or 600 nm is divided by the characteristic length of the cell (A/L). Normalized absorbance intensity per length of the cell (A/L) for *λ* = 1064 nm at various concentrations. **c** Photothermal rise curves as a function of concentrations under 1064 nm laser irradiation at 1.0 W/cm^2^. **d** Photothermal heating curves at different power densities with the concentration 50 µg/mL. **e** Temperature changes during four laser on-off cycles. **f** Photothermal conversion efficiency. Redline: Photothermal effects with 1064 nm laser irradiation for 15 min and then the temperature change with laser off. Blueline: Time constant (*τ*) obtained from the cooling profile

Although previous study has shown that FePS NSs could be used as photothermal agents in cancer therapy, [23] the NIR-II photothermal characteristics of FePS@PPF need to be further evaluated. Obvious absorption in the NIR-II region is observed from the UV-Vis-NIR spectra of FePS@PPF as a function of concentration (Fig. 2a). Both the absorbance of FePS@PPF at 1064 nm and 600 nm follows Lambert-Beer Law (R^2^ = 0.993 and 0.998, respectively). In consideration of deeper penetration depth and higher MPE of NIR-II light, we analyze the absorbance and photothermal conversion efficiency of FePS@PPF at 1064 nm. The extinction coefficient (*ε*) of the FePS@PPF is calculated to be 11.25 L g^− 1^ cm^− 1^ at 1064 nm by the Lambert-Beer law (Fig. 2b). Subsequently, FePS@PPF solutions are irradiated at 1.0 W/cm^2^ for 15 min and as demonstrated in Fig. 2c, there is no significant temperature changes for pure water, whereas remarkable concentration-depending temperature increment of FePS@PPF is observed under irradiation, indicating that FePS@PPF can effectively translate laser energy into heat. The photothermal properties of FePS@PPF also follow a power density-dependent trend (Fig. 2d). Moreover, no significant deterioration is detected after four cycles of heating and cooling (Fig. 2e) and the UV-Vis-NIR absorbance is not changed after irradiation (Additional file 1: Fig. S3), reflecting good photostability of FePS@PPF. Furthermore, the photothermal conversion efficiency of FePS@PPF is 47.1% under 1.0 W/cm^2^ (Fig. 2f), which is higher than that of many reported NIR-II photothermal agents,[32–36] indicating potential superiority for PTT.

### In vitro synergistic anti-tumor effects

**Fig. 3:**
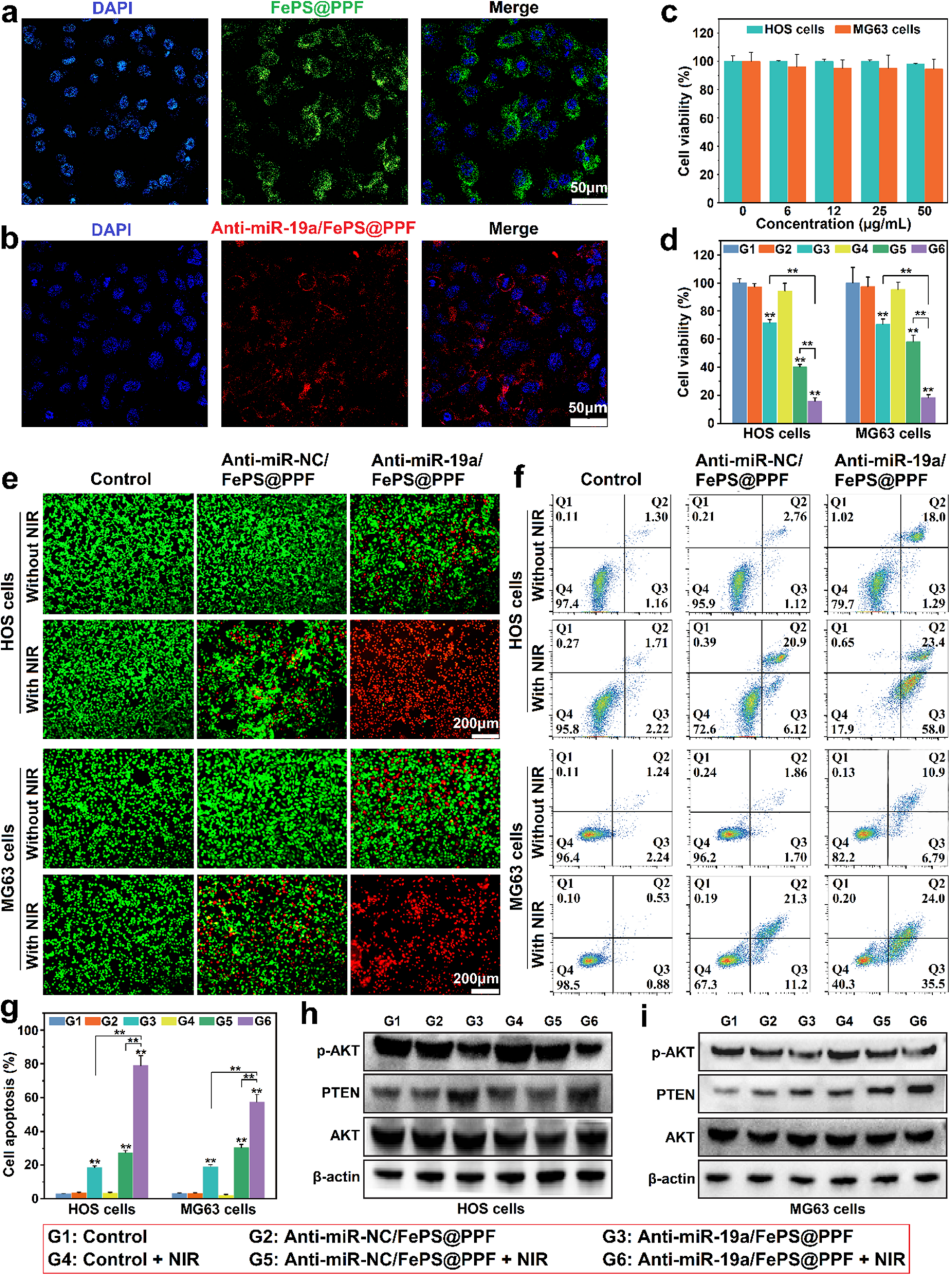
In vitro anti-tumor effect of anti-miRNA/FePS@PPF. **a** Images of HOS cells treated with FITC-labeled FePS@PPF and **b** Cy5.5-anti-miR-19a/FePS@PPF for 6 h (scale bar is 50 μm) obtained from confocal microscope. **c** Relative cell viabilities after incubation with FePS@PPF with various concentrations at 48 h. **d** Relative cell viability in the group of PBS (Control), anti-miR-NC/FePS@PPF and anti-miR-19a/FePS@PPF with or without NIR (FePS@PPF concentration of 25 µg/mL). **e** Fluorescence live (green)/dead (red) images (scale bar is 200 μm). **f** Flow cytometry analysis of apoptotic/necrotic cells and **g** the statistics of relative percentage of tumor cell apoptosis. **h**, **i** Western blot analysis of PTEN, AKT and p-AKT expression after different treatments for 48 h. **p < 0.01, G3, G5 and G6 compared to Control (G1)

Competent intracellular uptake of nanomaterials is the prerequisite against tumor cells. To visualize the intracellular uptake, FePS@PPF and anti-miR-19a are respectively labeled with FITC and Cy5.5 and confocal laser scanning microscope (CLSM) analysis is conducted after incubation with HOS cells. The fluorescence spectra of anti-miR-19a/FePS@PPF is similar as that of free anti-miR-19a and there is a good linear relationship between fluorescence intensity and concentration of anti-miR-19a, confirming the successful loading of anti-miR-19a (Additional file 1: Fig. S4). Compared to the control groups, the FePS@PPF-treated cells exhibit a significant amount of trapped particle (Additional file 1: Fig. S5). As shown in Fig. 3a, bright green is observed around the nuclei, implying the effective internalization of FePS@PPF. And the cells treated with anti-miR-19a/FePS@PPF are stained red implying efficient transportation of anti-miR-19a by the nanocarrier, FePS@PPF (Fig. 3b). Moreover, no significant cytotoxicity is detected from both the HOS cells and MG63 cells after incubated with FePS@PPF even at 50 µg/mL (Fig. 3c). These results demonstrate that FePS@PPF has good biocompatibility and can be efficiently internalized by osteosarcoma cells due to the surface modification of folic acid.

**Fig. 4:**
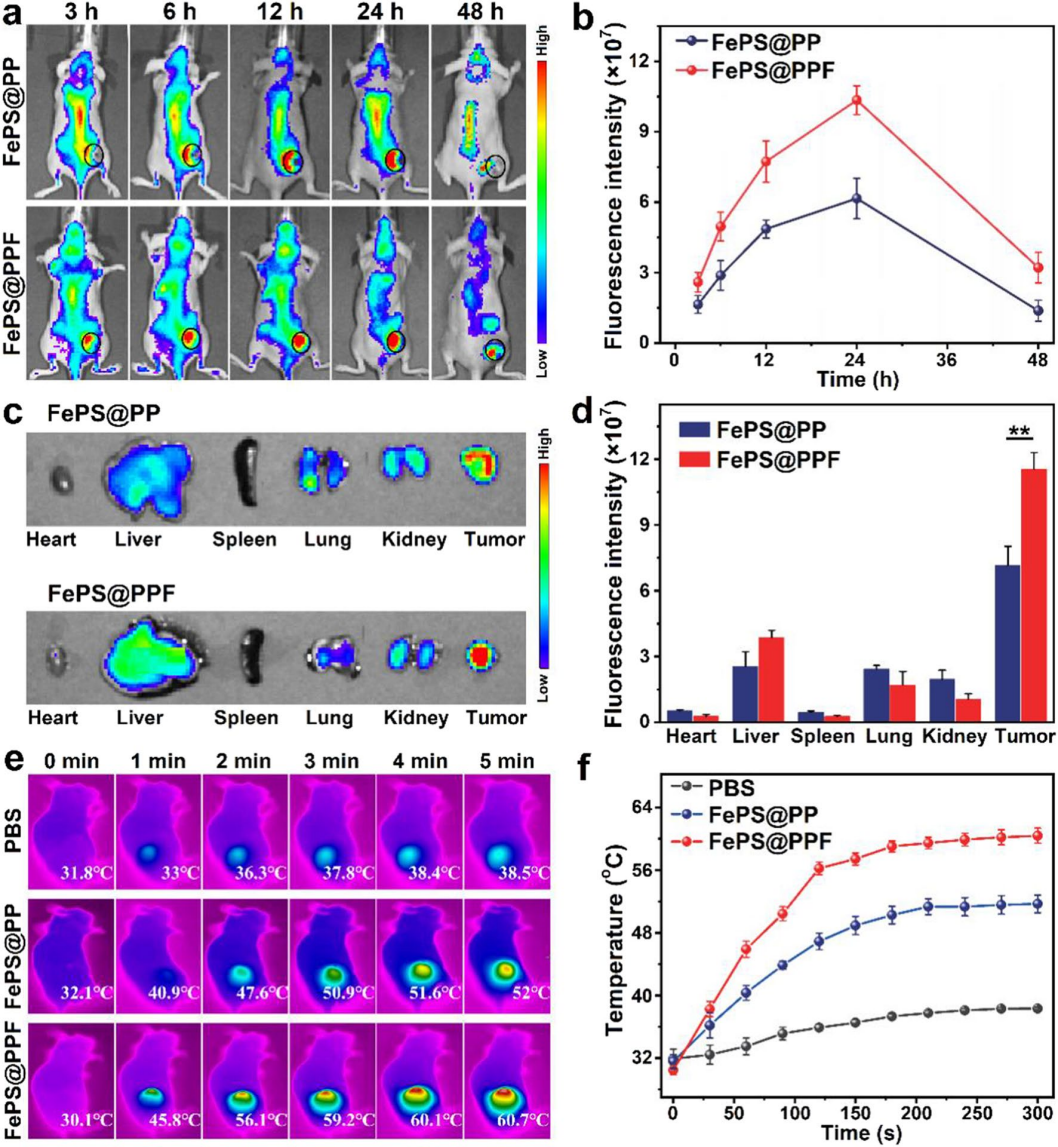
In vivo biodistributions of FePS@PPF. **a** In vivo fluorescence images of mice with HOS tumors (marked by ellipses) after injection of Cy5.5-labeled FePS NSs without (FePS@PP) or with folic acid modification (FePS@PPF). **b** Fluorescence intensity of tumor sites obtained by Living Image software. **c** Ex vivo fluorescence images. **d** Average fluorescence intensity of main organs and tumor, **p < 0.01. **e** Infrared thermographic images and **f** Temperature changes of tumor site under laser irradiation (1064 nm, 1.0 W/cm^2^) at 24 h after respectively intravenous infusion of PBS, FePS@PP and FePS@PPF

The in vitro therapeutic efficacy of PTT and gene therapy is subsequently investigated under 1064 nm laser irradiation for 10 min at 1.0 W/cm^2^ (Fig. 3d). As CCK-8 assay in Fig. 4d shown, there is no apparent cell viability change in negative control groups that the cells are treated with PBS, PBS + NIR and anti-miR-NC/FePS@PPF. After treatment with anti-miR-19a/FePS@PPF and anti-miR-NC/FePS@PPF + NIR, the cell viabilities are significantly lower than that of control groups, suggesting considerable anti-cancer effect of monotherapy by gene therapy or PTT, respectively. However, the viability of anti-miR-19a/FePS@PPF + NIR is further much lower than that of the monotherapy groups (gene therapy by anti-miR-19a/FePS@PPF or PTT by anti-miR-NC/FePS@PPF + NIR). The live/dead co-staining by Calcein-AM/PI reveals that nearly all of the cells are killed after treatment with anti-miR-19a/FePS@PPF + NIR in comparison to other groups (Fig. 3e). These results indicate the synergistic anti-cancer effect of PTT and gene therapy mediated by the multifunctional anti-miR-19a/FePS@PPF nanoplatform.

The mechanisms underlying this synergistic anti-cancer effect are demonstrated by flow cytometry as well as Western blot. The Annexin V-FITC/PI co-staining assay in Fig. 3f and g shows that cell apoptosis obviously occurs in anti-miR-19a/FePS@PPF group and anti-miR-NC/FePS@PPF + NIR group, and the total apoptosis ratios in HOS and MG63 cells are 19.3%, 27.0% and 17.7%, 32.5%, respectively. Whereas the apoptotic cells occupy as high as 81.4% and 59.5% in anti-miR-19a/FePS@PPF + NIR group for HOS and MG63 cells, respectively. Thus, there is the distinct synergy between gene and photothermal therapy against osteosarcoma in vitro. It is well known that miR-19a can target to PTEN gene, which actives the phosphorylation of AKT and further promotes osteosarcoma growth; while anti-miR-19a reverses this effect.[37–39] After transfected HOS cells and MG63 cells with anti-miR-19a/FePS@PPF, Western blot assay is conducted and the results show that expression of PTEN is up-regulated and p-AKT is down-regulated compared to anti-miR-NC/FePS@PPF group with or without NIR irradiation (Fig. 3h, i). The above-mentioned results confirm that anti-miR-19a/FePS@PPF participates in the PTEN/AKT pathway for therapy of osteosarcoma. Therefore, FePS@PPF could serve as an efficient multiplatform to deliver anti-miR-19a into osteosarcoma cells and kill tumor cells by synergetic gene-photothermal therapy after exposure to NIR-II laser.

### In vivo biodistribution and tumor targeting

Before evaluating the therapeutic effects, the murine tumor targeting ability of FePS@PPF is investigated. The Cy5.5-labeled FePS NSs without (FePS@PP) or with folic acid modification (FePS@PPF) are intravenously administrated into mice bearing HOS tumors to record the distribution of materials over time using IVIS fluorescence imaging system. As shown in Fig. 4a, the fluorescence signals are distributed over the entire mouse administrated with FePS@PP during 24 h and then decrease within 48 h. In contrast, more tumor accumulation of FePS@PPF is observed than that of FePS@PP. Quantitative analysis indicates that the fluorescence intensity of tumor sites reaches to the maximum at 24 h for both groups (Fig. 4b). Therefore, mice are sacrificed for ex vivo fluorescence imaging of the major organs and tumor tissues after 24 h (Fig. 4c, d). The fluorescence intensity of tumor in FePS@PPF-treated mice is significantly higher than that in FePS@PP-treated mice. As a conclusion, FePS@PPF exhibits excellent tumor targeting specificity due to the interaction between folic acid and folate receptor overexpressed in tumor.

The enhanced tumor accumulation of FePS@PPF is expected to facilitate photothermal performance. As shown in Fig. 4e, f, the temperature of the FePS@PP group rises by 19.9 ^o^C after irradiation of 1064 nm laser (5 min), while the FePS@PPF group significantly increases by 30.6 ^o^C. Obviously, FePS@PPF exhibits improved in vivo photothermal performances resulting from the tumor targeting capability. These results show the great potential application of FePS@PPF as photothermal agents in tumor treatment. However, the photothermal effect of FePS@PPF attenuates with the increase of thickness of tumor tissue (Additional file 1: Fig. S6), which may result in incomplete treatment and recurrence of tumor. Hence, it is highly desirable to develop a multifunctional nanoplatform based on the synergistic effect of PTT and other therapy for efficient treatment of osteosarcoma.

### In vivo synergistic anti-tumor effects

**Fig. 5:**
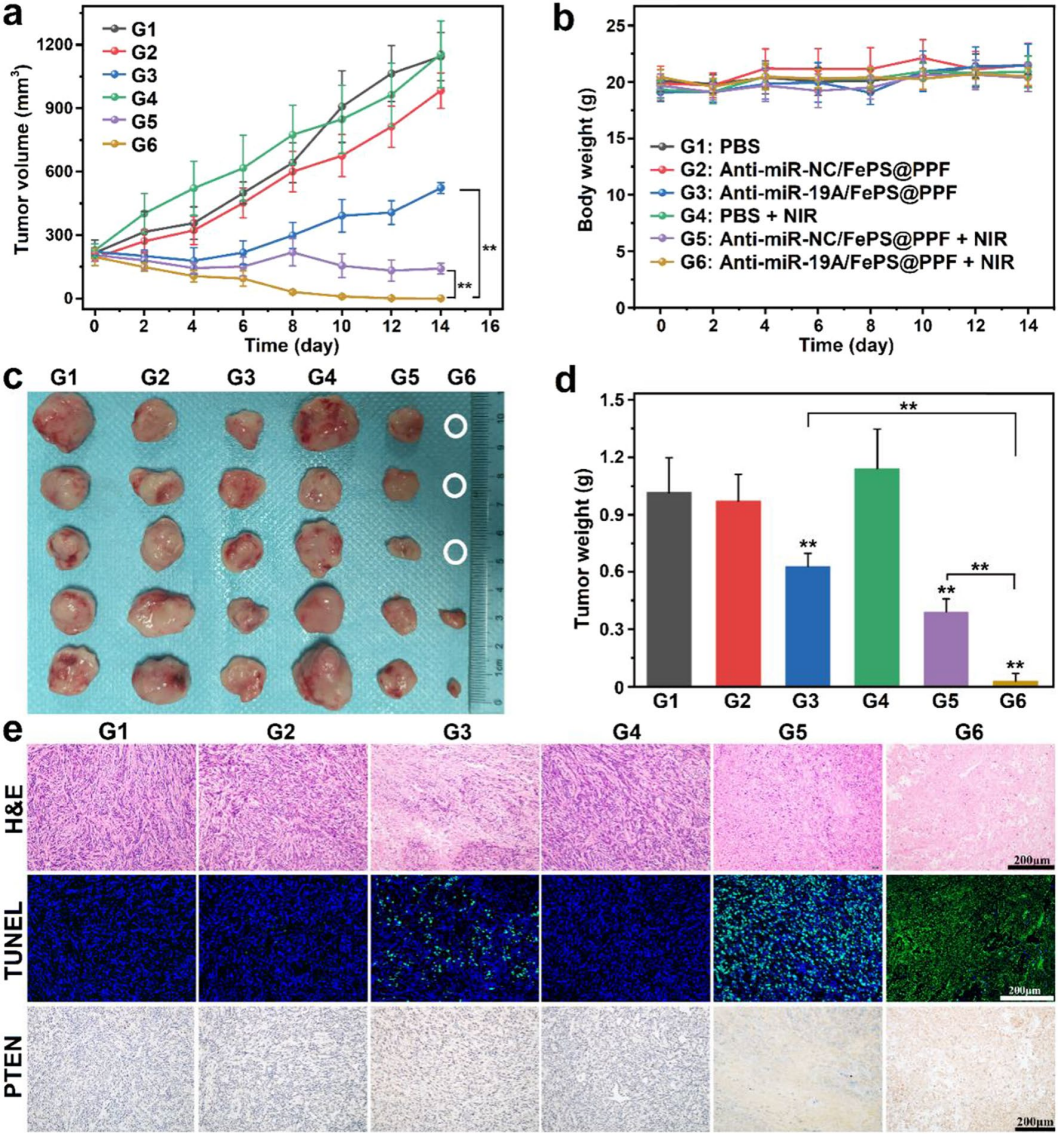
In vivo anti-tumor activity of anti-miR-19a/FePS@PPF in HOS tumor-bearing mice. **a** Changes of tumor volume and **b** Body weights of mice at different time points after various treatments. **c** Photographs of tumors and **d** Tumor weights at day 14. **e** H&E, TUNEL and IHC staining of the tumor sections at the end of treatment. n = 5, **p < 0.01, G3, G5 and G6 compared to PBS (G1)

Then the anti-miR-19a/FePS@PPF-mediated gene-photothermal therapy in vivo is assessed by continuously measuring tumor volumes after administrated with different formulations with or without 1064 nm laser irradiation. As shown in Fig. 5a, tumors in the Control groups (PBS, PBS + laser, and anti-miR-NC/FePS@PPF group) grow rapidly and the tumor volumes reach to 1144.2, 1155.2, and 983.2 mm^3^ after 14 days, respectively, indicating negligible phototoxicity of 1064 nm laser and good biocompatibility of FePS@PPF nanocarrier. Treatments of anti-miR-19a/FePS@PPF and anti-miR-NC/FePS@PPF + NIR reduce tumor volume to 522.5 and 141.1 mm^3^, respectively, demonstrating that monotherapy of gene therapy or PPT could moderately inhibit tumor growth. The tumor volume of mice treated with anti-miR-19a/FePS@PPF + NIR is only 0.17 mm^3^ and some of the tumors are completely cleared (Fig. 5c), showing synergistic anti-cancer effect by combination of gene therapy and PPT. Tumors are removed and weighted after treatment. The tumor weight of anti-miR-19a/FePS@PPF + NIR group is significantly lower than that of other groups (Fig. 5d). Besides, there is no change in body weight for all groups due to the good biosafety of this combined therapy (Fig. 5b). These above results prove that the combined gene-photothermal therapy mediated by anti-miR-19a/FePS@PPF remarkably enhances in vivo therapeutic effect against osteosarcoma.

The limited penetration depth of NIR laser leads to uneven heat distribution in the tumor area and further results in incomplete treatment and possible recurrence. On the other hand, critical obstacles accompanied with anti-miR-19a remain to be overcome such as poor stability in vivo, high rate of blood clearance and poor aggregation to the tumor tissues. Intriguingly, the integration of PTT and gene therapy can complement each other to achieve synergistic therapeutic effect. In detail, anti-miR-19a is protected and target delivered to tumor site by FePS@PPF. Subsequently, under NIR irradiation, most of the tumor tissues are ablated by the photothermal effect and the residual tumor cells are further cleared by anti-miR-19a. As shown in Fig. 5, the anti-tumor effects in combined treatment group is significantly improved compared to single PTT or gene therapy.

To verify the superior anti-cancer effects, H&E staining and TUNEL assay are performed to examine the tumor sections at the end of treatment. As illustrated in Fig. 5e, vigorous growth in the Control groups is observed from the intact shape and tight arrangement of tumor cells. Cell necrosis, fragmentation and lysis occur in anti-miR-19a/FePS@PPF and anti-miR-NC/PPF + NIR group, while anti-miR-19a/FePS@PPF + NIR group appeared even more serve necrosis and morphological changes of tumors. In addition, the most of apoptosis (green fluorescence) in the anti-miR-19a/FePS@PPF + NIR group is observed from TUNEL assay than other groups. In osteosarcoma cells, PTEN is a well-known tumor suppressor that negatively regulates the phosphorylation of p-AKT, and miR-19a could regulate the expression of PTEN by targeting degradation [37, 40]. To clarify whether the osteosarcoma suppression mediated by anti-miR-19a/FePS@PPF was associated with PTEN, immunohistochemistry (IHC) analysis is conducted and the results revealed that the PTEN protein level in tumor tissues is significantly increased by anti-miR-19a/FePS@PPF whether with or without NIR irradiation compared to other groups (Fig. 5e). As previously reported, the activity of siRNA is significantly decreased by ultrahigh temperature sterilization (135 ^o^C), while pasteurization (85 ^o^C) had no significant effect on the content of miRNA [41]. In our study, the highest temperature for in vitro and in vivo studies reached to around 60 ^o^C, which is expected to have no influence on the activity of siRNA. These results confirm the excellent antitumor activity of the combined gene-photothermal treatment by the multifunctional anti-miR-19a/FePS@PPF nanoplatform.

### In vivo biodegradation and biosafety evaluation

**Fig. 6:**
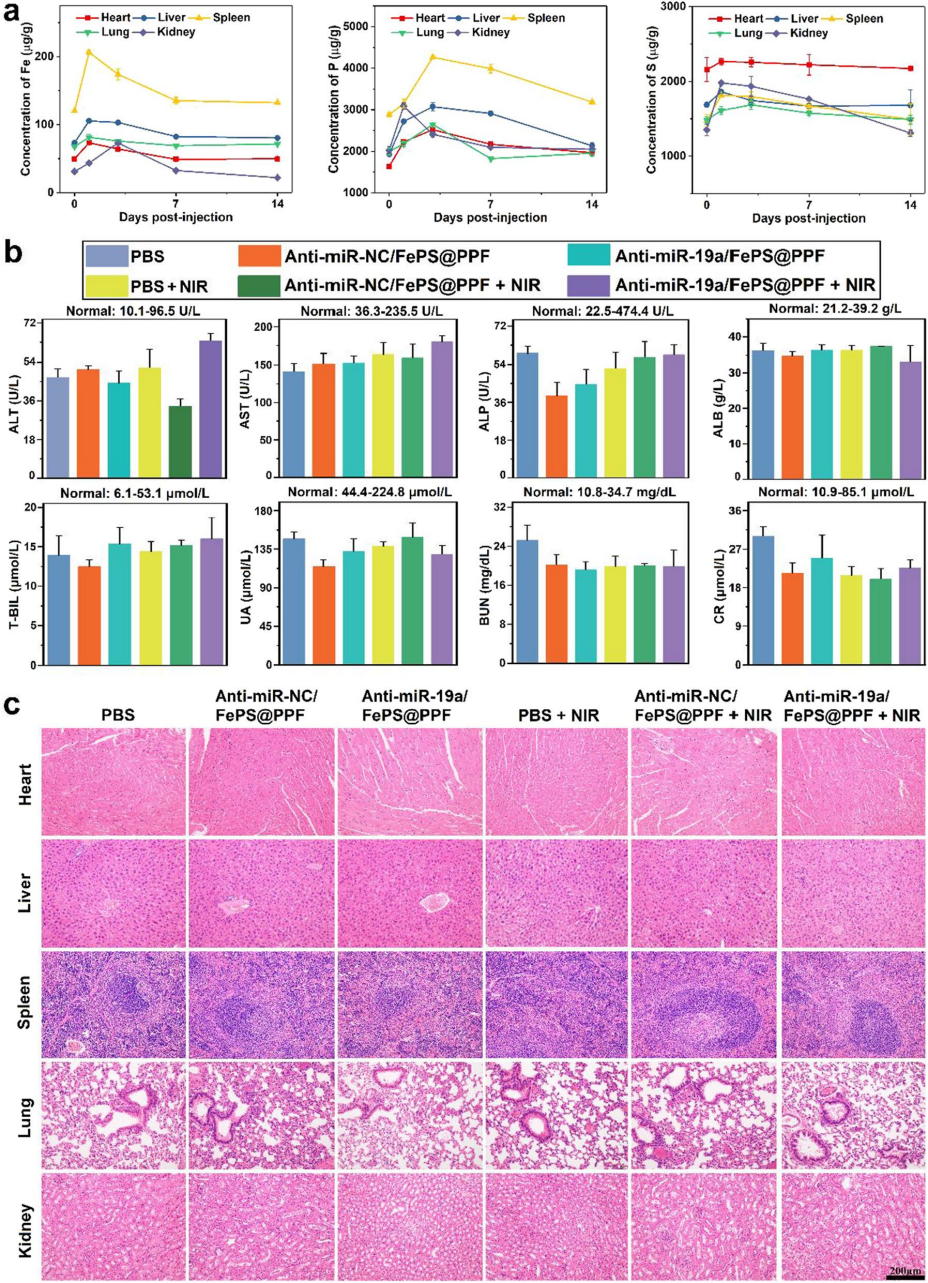
In vivo biodegradation and biosafety of FePS@PPF. **a** Concentration curves of Fe, P and S in different organs at various time points post-injection. **b** Blood biochemical analysis and (c) H&E staining of major organs at 14 days after various treatments

Biosafety and biodegradation are critical issues in clinical applications because nanomaterials that could not be cleared by body can cause potential health harm. Here, the FePS@PPF nanocarrier is administrated intravenously into tumor-bearing mice (10 mg/kg), which are sacrificed at 1, 3, 7, 14 days post-injection. The major organs including heat, liver, lung, spleen and kidney are collected and the Fe, P and S concentrations are determined by ICP-OES to evaluate the biodegradation paths of FePS@PPF. As shown in Fig. 6a, all of the three elements increase after 1-day post-injection and gradually decrease from day 3. The elemental levels at day 14 are nearly the same as as those of day 0 (before injection of FePS@PPF), indicating FePS@PPF is eliminated at the end of treatment. The excellent biodegradation of FePS@PPF make it a desirable candidate for long-term use in biomedicine. Although many nanomaterials possess good photothermal properties in the NIR-II range, most of them are not biodegradable and cannot be excreted naturally to avoid potential long-term toxicity in vivo. Therefore, the major advantage of our work lies in proposing a biodegradable nanotherapeutic platform based on FePS NSs and demonstrating it as a highly effective and safe tumor therapeutic agent in the NIR-II region.

After treatment for 14 days, blood biochemical analysis is carried out to assess liver (ALT, AST, ALP, ALB, and T-BIL refer to alanine transaminase, aspartate transaminase, alkaline phosphatase, albumin, and total bilirubin, respectively) and renal (UA, BUN, and CR refer to uric acid, blood urea nitrogen, and creatinine) functions. The level of these biomarkers are normal, suggesting no hepatic and renal dysfunction are occurred in all groups (Fig. 6b). In addition, H&E staining is performed for the major organs and the results show that the injection of FePS@PPF materials cause no significant damage to the main organs compared with the PBS group (Fig. 6c). Above results indicate the potential of FePS@PPF as a powerful therapeutic nanoparticle with good biosafety.

## Conclusion

In summary, the folic acid-conjugated and anti-miR-19a-loaded FePS NSs (anti-miR-19a/FePS@PPF) are developed to integate the following properties for osteosarcoma therapy: tumor-targeted gene therapy and NIR-II-laser responsive PTT. It is shown that anti-miR-19a/FePS@PPF can be effective uptake and efficiently deliver anti-miR-19a into osteosarcoma cells. After intravenous administration, FePS@PPF specifically aggregates at tumor sites and improve the photothermal effects of tumor after exposure to 1064 nm laser. Both the in vitro and in vivo experiments demonstrate that single PTT mediated by anti-miR-NC/FePS@PPF (with NIR) or gene therapy mediated by anti-miR-19a/FePS@PPF (without NIR) can only moderately inhibit tumor cell growth. More importantly, anti-miR-19a/FePS@PPF under NIR-II laser irradiation lead to better treatment against osteosarcoma. Besides, the FePS@PPF nanoplatform possesses excellent biodegradation and biosafety in vivo. Our work provides strong evidence that the synergistic therapeutic strategy of anti-miR-19a/FePS@PPF can be utilized for osteosarcoma treatment with minimal side effects.

## Supplementary information

**Additional file 1:** **Fig. S1** Size distribution analyzed by DLS.AFMimage of FePS NSs. **Fig. S2 **Digital images of FePSNSs and FePS@PPF dispersed in water with different periods of time. **Fig. S3** The UV-Vis-NIR absorbance and digital images ofFePS@PPF before/after four laser on/off cycles. **Fig. S4** The fluorescence detection of Cy5.5-labeledanti-miR-19a.Emissionspectrum of Cy5.5-labeled anti-miR-19a and Cy5.5-labeled anti-miR-19a/FePS@PPF at 635 nm of excitation. Thefluorescence intensity of Cy5.5-labeledanti-miR-19a at different concentration. **Fig. S5** Bright-field imagesof HOS cells and MG63 cells with or without treatment of FePS@PPF. Scale bar is100 µm. **Fig. S6** Digital imagesof different tumor thicknesses.Temperature of FePS@PPFafter the irradiation of 1064 nm laserfor 10 min.

## Data Availability

The authors declare that all data supporting the results in this study are available within the paper and its Supplementary Information.

## References

[1] Wilhelm M, Dirksen U, Bielack S, Whelan J, Lewis I, Jürgens H (2014). ENCCA WP17-WP7 consensus paper on teenagers and young adults (TYA) with bone sarcomas. Ann Oncol.

[2] Isakoff MS, Bielack SS, Meltzer P, Gorlick R (2015). Osteosarcoma: current treatment and a collaborative pathway to Success. J Clin Oncol.

[3] Levesque J-P, Winkler IG (2013). It takes nerves to recover from chemotherapy. Nat Med.

[4] Cleeland CS, Allen JD, Roberts SA, Brell JM, Giralt SA, Khakoo AY (2012). Reducing the toxicity of cancer therapy: recognizing needs, taking action. Nat Rev Clin Oncol.

[5] Peer D, Karp JM, Hong S, Farokhzad OC, Margalit R, Langer R (2020). Nanocarriers as an emerging platform for cancer therapy. Nano-Enabled Med Appl.

[6] Gill J, Gorlick R (2021). Advancing therapy for osteosarcoma. Nat Rev Clin Oncol.

[7] Michael JV, Wurtzel JGT, Mao GF, Rao AK, Kolpakov MA, Sabri A (2017). Platelet microparticles infiltrating solid tumors transfer miRNAs that suppress tumor growth. Blood.

[8] Orso F, Quirico L, Dettori D, Coppo R, Virga F, Ferreira LC (2020). Role of miRNAs in tumor and endothelial cell interactions during tumor progression. Seminars Cancer Biol.

[9] Chong ZX, Yeap SK, Ho WY (2021). Unraveling the roles of miRNAs in regulating epithelial-to-mesenchymal transition (EMT) in osteosarcoma. Pharmacol Res.

[10] Shan HJ, Zhu LQ, Yao C, Zhang ZQ, Liu YY, Jiang Q (2021). MAFG-driven osteosarcoma cell progression is inhibited by a novel miRNA miR-4660. Mol Ther-Nucl Acids.

[11] Bravo V, Rosero S, Ricordi C, Pastori RL (2007). Instability of miRNA and cDNAs derivatives in RNA preparations. Biochem Biophy Res Commun.

[12] Chen Y, Gao DY, Huang L (2015). In vivo delivery of miRNAs for cancer therapy: challenges and strategies. Adv Drug Deliver Rev.

[13] Grimm D, Streetz KL, Jopling CL, Storm TA, Pandey K, Davis CR (2006). Fatality in mice due to oversaturation of cellular microRNA/short hairpin RNA pathways. Nature.

[14] Pecot CV, Calin GA, Coleman RL, Lopez-Berestein G, Sood AK (2011). RNA interference in the clinic: challenges and future directions. Nat Rev Cancer.

[15] Zhong Z, Liu C, Xu Y, Si W, Wang W, Zhong L (2022). γ-Fe2O3 loading mitoxantrone and glucose oxidase for pH‐responsive chemo/chemodynamic/photothermal synergistic cancer therapy. Adv Healthc Mater.

[16] Guo R, Wang S, Zhao L, Zong Q, Li T, Ling G (2022). Engineered nanomaterials for synergistic photo-immunotherapy. Biomaterials.

[17] Zeng W, Zhang H, Deng Y, Jiang A, Bao X, Guo M (2020). Dual-response oxygen-generating MnO2 nanoparticles with polydopamine modification for combined photothermal-photodynamic therapy. Chem Eng J.

[18] Tao W, Cheng X, Sun D, Guo Y, Wang N, Ruan J (2022). Synthesis of multi-branched au nanocomposites with distinct plasmon resonance in NIR-II window and controlled CRISPR-Cas9 delivery for synergistic gene-photothermal therapy. Biomaterials.

[19] Xu J, Chen L, Ding S, Dai X, Dai Y, Chen Y (2023). Self-generated Schottky barriers in niobium carbide MXene nanocatalysts for theory-oriented sonocatalytic and NIR-II photonic hyperthermia tumor therapy. Nano Today.

[20] Zhang M, Lin J, Jin J, Yu W, Qi Y, Tao H (2021). Delivery of siRNA using functionalized gold nanorods enhances anti-osteosarcoma efficacy. Front Pharmacol.

[21] Kou Z, Wang X, Yuan R, Chen H, Zhi Q, Gao L (2014). A promising gene delivery system developed from PEGylated MoS2 nanosheets for gene therapy. Nanoscale Res Lett.

[22] Zhang C, Yong Y, Song L, Dong X, Zhang X, Liu X (2016). Multifunctional WS2@ poly (ethylene imine) nanoplatforms for imaging guided gene-photothermal synergistic therapy of Cancer. Adv Healthc Mater.

[23] Zhang Q, Guo Q, Chen Q, Zhao X, Pennycook SJ, Chen H (2020). Highly efficient 2D NIR-II photothermal agent with fenton catalytic activity for cancer synergistic photothermal-chemodynamic therapy. Adv Sci.

[24] Fang X, Wu X, Li Z, Jiang L, Lo WS, Chen G (2021). Biomimetic Anti-PD‐1 peptide‐loaded 2D FePSe3 nanosheets for efficient photothermal and enhanced Immune Therapy with Multimodal MR/PA/Thermal Imaging. Adv Sci.

[25] Huang K, Zhang Y, Lin J, Huang P (2019). Nanomaterials for photoacoustic imaging in the second near-infrared window. Biomater Sci.

[26] Lin H, Gao S, Dai C, Chen Y, Shi J (2017). A two-dimensional biodegradable niobium carbide (MXene) for photothermal tumor eradication in NIR-I and NIR-II biowindows. J Am Chem Soc.

[27] Thayanithy V, Dickson EL, Steer C, Subramanian S, Lou E (2014). Tumor-stromal cross talk: direct cell-to-cell transfer of oncogenic microRNAs via tunneling nanotubes. Transl Res.

[28] Zou Q, Xiao X, Liang Y, Peng L, Guo Z, Li W (2018). miR-19a-mediated downregulation of RhoB inhibits the dephosphorylation of AKT1 and induces osteosarcoma cell metastasis. Cancer Lett.

[29] Xing S, Qu Y, Li C, Huang A, Tong S, Wu C (2019). Deregulation of lncRNA-AC078883.3 and microRNA-19a is involved in the development of chemoresistance to cisplatin via modulating signaling pathway of PTEN/AKT. J Cell Physiol.

[30] Zhao W, Li A, Zhang A, Zheng Y, Liu J (2018). Recent advances in functional-polymer‐decorated transition‐metal nanomaterials for Bioimaging and Cancer Therapy. ChemMedChem.

[31] Yang G, Phua SZF, Bindra AK, Zhao Y (2019). Degradability and clearance of inorganic nanoparticles for biomedical applications. Adv Mater.

[32] Huang C, Sun Z, Cui H, Pan T, Geng S, Zhou W (2019). InSe nanosheets for efficient NIR-II-responsive drug release. ACS Appl Mater Inter.

[33] Guo B, Sheng Z, Hu D, Liu C, Zheng H, Liu B (2018). Through scalp and Skull NIR-II photothermal therapy of deep orthotopic brain tumors with precise photoacoustic imaging guidance. Adv Mater.

[34] Cheng Q, Tian Y, Dang H, Teng C, Xie K, Yin D (2022). Antiquenching macromolecular NIR-II probes with high-contrast brightness for imaging-guided Photothermal Therapy under 1064 nm irradiation. Adv Healthc Mater.

[35] Yin H, Guan X, Lin H, Pu Y, Fang Y, Yue W (2020). Nanomedicine-enabled photonic thermogaseous cancer therapy. Adv Sci.

[36] Wu C, Wang D, Cen M, Cao L, Ding Y, Wang J (2020). Mitochondria-targeting NO gas nanogenerator for augmenting mild photothermal therapy in the NIR-II biowindow. Chem Commun.

[37] Luo T, Zhou X, Jiang E, Wang L, Ji Y, Shang Z (2021). Osteosarcoma cell-derived small extracellular vesicles enhance osteoclastogenesis and bone resorption through transferring MicroRNA-19a-3p. Front Oncol.

[38] Zhao D, Chen Y, Chen S, Zheng C, Hu J, Luo S (2017). MiR-19a regulates the cell growth and apoptosis of osteosarcoma stem cells by targeting PTEN. Tumour Biol.

[39] Li X, Sun XH, Xu HY, Pan HS, Liu Y, He L (2019). Circ_ORC2 enhances the regulatory effect of miR-19a on its target gene PTEN to affect osteosarcoma cell growth. Biochem Biophy Res Commun.

[40] Zheng C, Tang F, Min L, Hornicek F, Duan Z, Tu C (2020). PTEN in osteosarcoma: recent advances and the therapeutic potential. BBA-Rev Cancer.

[41] Zhang Y, Xu Q, Hou J, Huang G, Zhao S, Zheng N (2022). Loss of bioactive microRNAs in cow’s milk by ultra-high-temperature treatment but not by pasteurization treatment. J Sci Food Agr.

