# Supplementary material for: Biodegradable FePS3 nanoplatform for efficient treatment of osteosarcoma by combination of gene and NIR-II photothermal therapy
Source: J Nanobiotechnology. 2023 Jul 13;21:224. doi: 10.1186/s12951-023-01961-9 (PMC10347837; doi:10.1186/s12951-023-01961-9)
Supplement: Supplementary file 1 — Additional file 1: Fig. S1 Size distribution analyzed by DLS.AFMimage of FePS NSs. Fig. S2 Digital images of FePSNSs and FePS@PPF dispersed in water with different periods of time. Fig. S3 The UV-Vis-NIR absorbance and digital images ofFePS@PPF before/after four laser on/off cycles. Fig. S4 The fluorescence detection of Cy5.5-labeledanti-miR-19a.Emissionspectrum of Cy5.5-labeled anti-miR-19a and Cy5.5-labeled anti-miR-19a/FePS@PPF at 635 nm of excitation. Thefluorescence intensity of Cy5.5-labeledanti-miR-19a at different concentration. Fig. S5 Bright-field imagesof HOS cells and MG63 cells with or without treatment of FePS@PPF. Scale bar is100 µm. Fig. S6 Digital imagesof different tumor thicknesses.Temperature of FePS@PPFafter the irradiation of 1064 nm laserfor 10 min. [file 12951_2023_1961_MOESM1_ESM.docx]

Supporting Information

**Biodegradable FePS_3_ nanoplatform for efficient treatment of osteosarcoma by combination of gene and NIR-II photothermal therapy**

Tingting Luo^1†^, Mingyang Jiang^2^^†^, Ziqiang Cheng^3†^, Yuntao Lin^1^, Yuling Chen^1^, Zhenyu Zhang^2^, Jian Zhou^1^, Wenhua Zhou^2^, Xue-Feng Yu^2^, Shuchun Li^1*^, Shengyong Geng^2*^, and Hongyu Yang^1*^

^1^ Guangdong Provincial High-level Clinical Key Specialty, Guangdong Province Engineering Research Center of Oral Disease Diagnosis and Treatment, The Institute of Stomatology, Peking University Shenzhen Hospital, Shenzhen Peking University-The Hong Kong University of Science and Technology Medical Center, Guangdong province, China

^2^ Materials and Interfaces Center, Shenzhen Institutes of Advanced Technology, Chinese Academy of Sciences, Shenzhen 518055, China

^3^ Department of Applied Physics, School of Science, East China Jiaotong University, Nanchang 330013, China

* Corresponding authors:

Shengyong Geng, Email: [sy.geng@siat.ac.cn](mailto:sy.geng@siat.ac.cn)

Shuchun Li, Email: [lisc56@163.com](mailto:lisc56@163.com)

Hongyu Yang, Email: [hyyang192@hotmail.com](mailto:hyyang192@hotmail.com)

^†^ These authors contributed equally to this work.

**
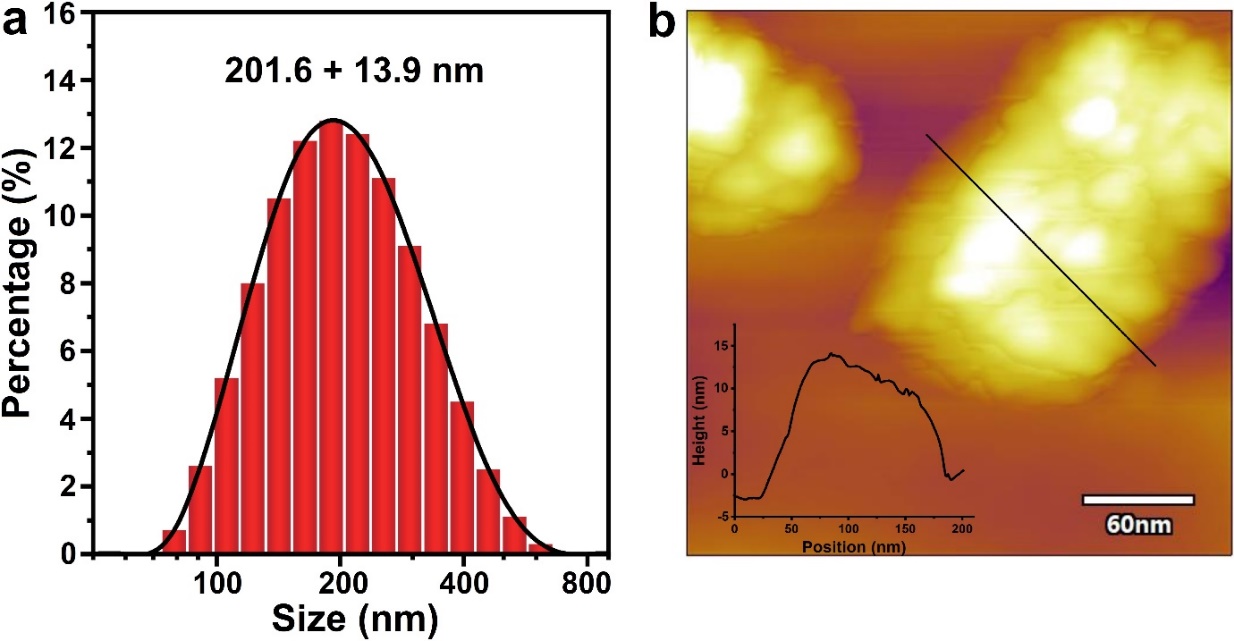
**

Fig. S1 (a) Size distribution analyzed by DLS. (b) AFM image of FePS NSs.


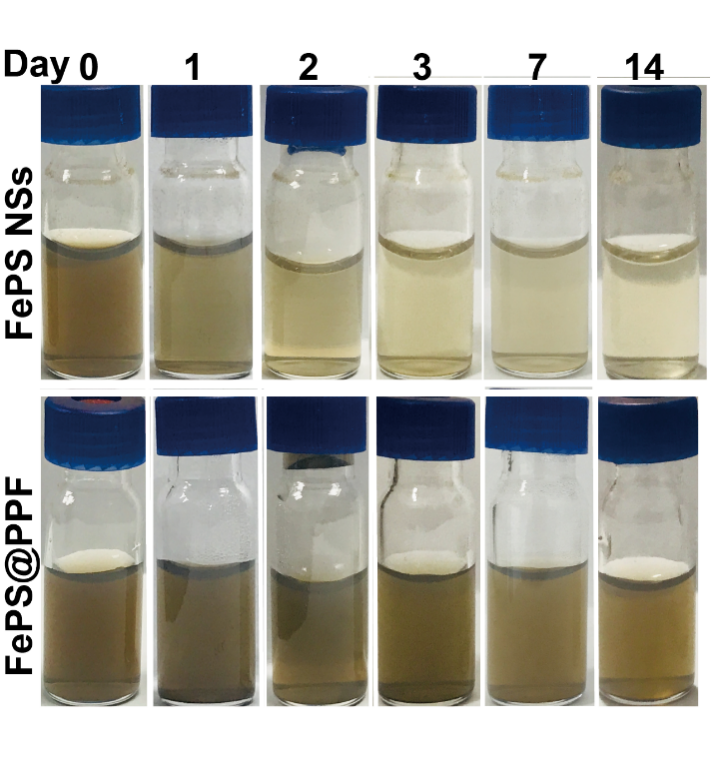


**Fig. S2** Digital images of FePS NSs and FePS@PPF dispersed in water with different periods of time.


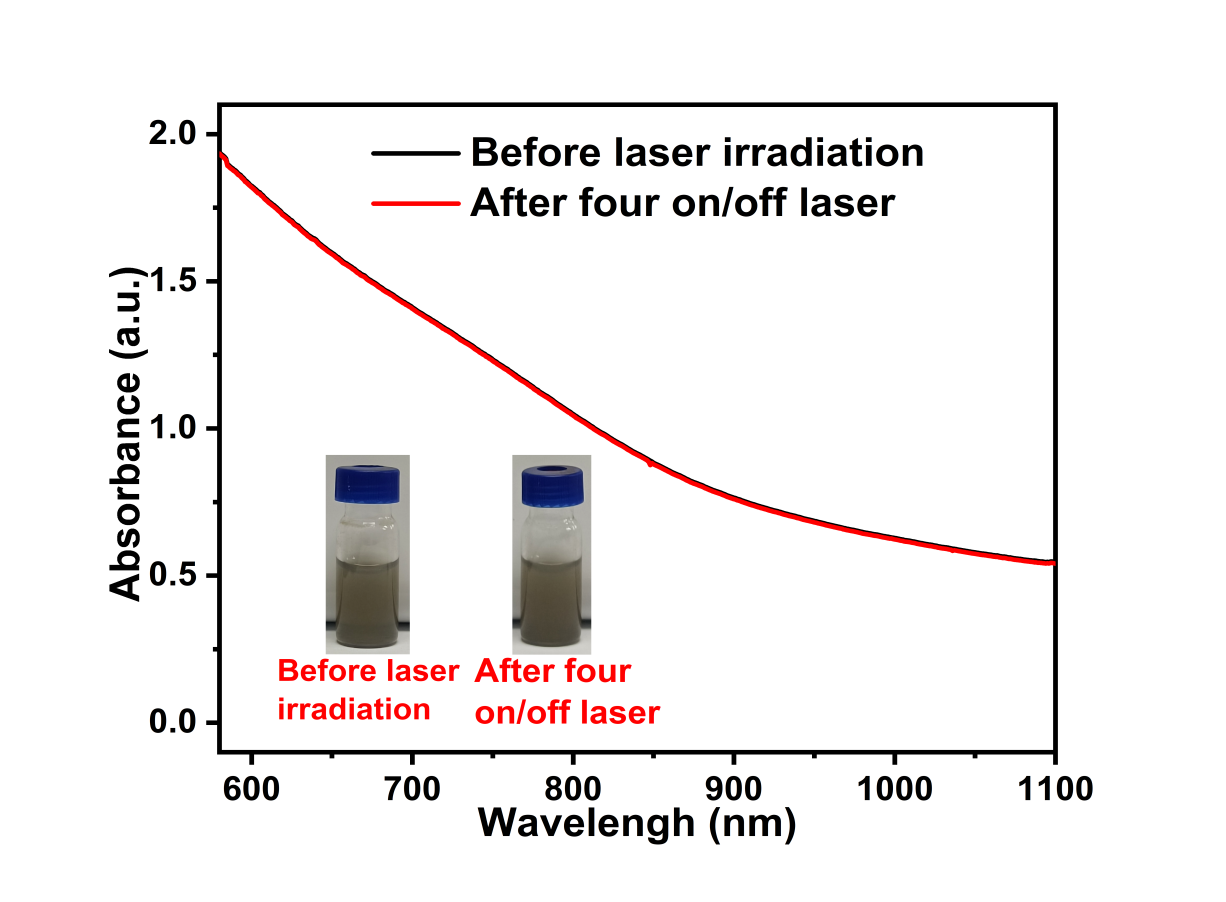


**Fig. S3** The UV-Vis-NIR absorbance and digital images of FePS@PPF before/after four laser on/off cycles.


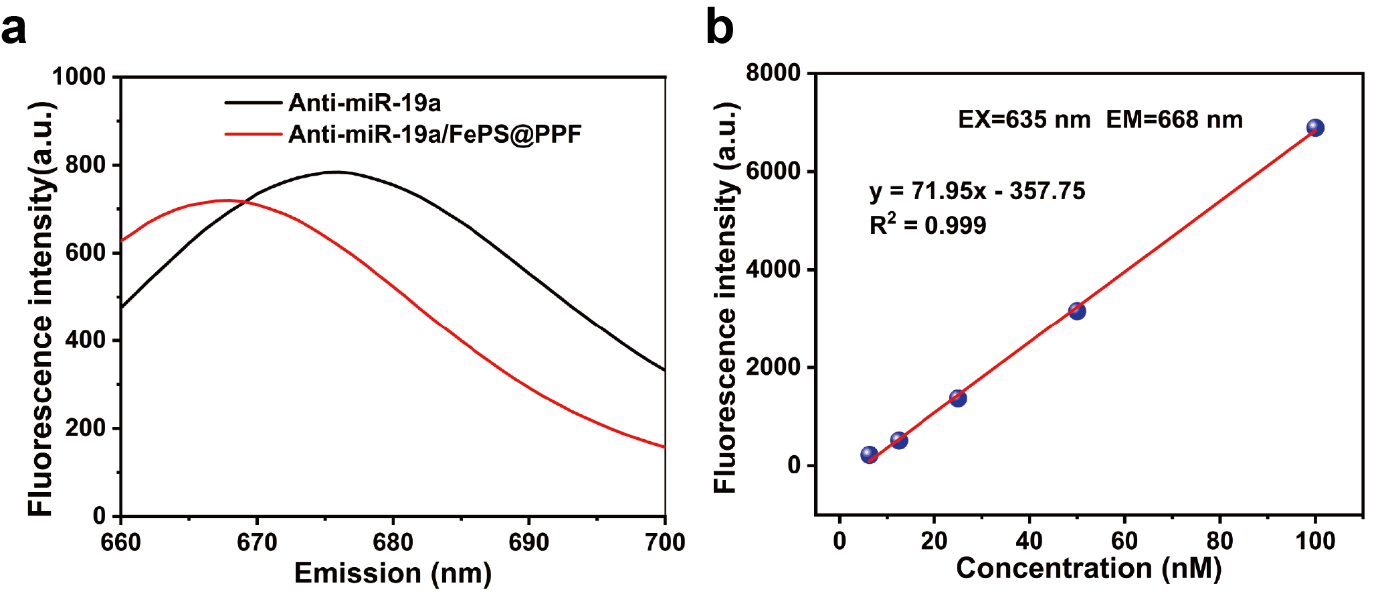


**Fig. S4** The fluorescence detection of Cy5.5-labeled anti-miR-19a. (a) Emission (EM) spectrum of Cy5.5-labeled anti-miR-19a and Cy5.5-labeled anti-miR-19a/FePS@PPF at 635 nm of excitation. (b) The fluorescence intensity of Cy5.5-labeled anti-miR-19a at different concentration (EX = 635 nm, EM = 668 nm).


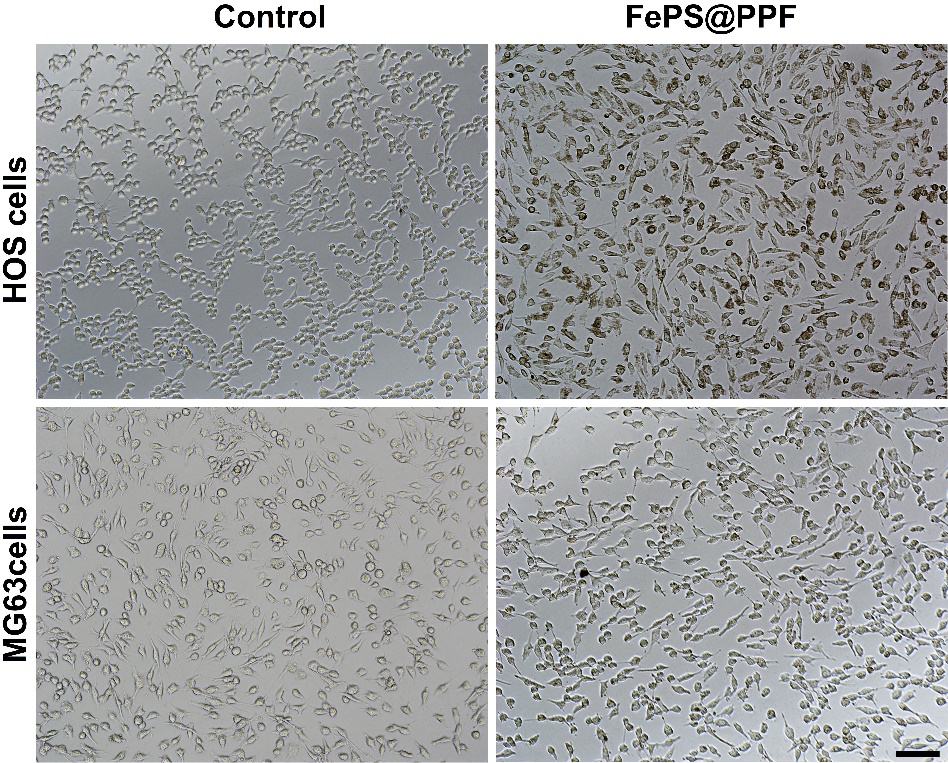


**Fig. S5** Bright-field images of HOS cells and MG63 cells with or without treatment of FePS@PPF. Scale bar is 100 µm.


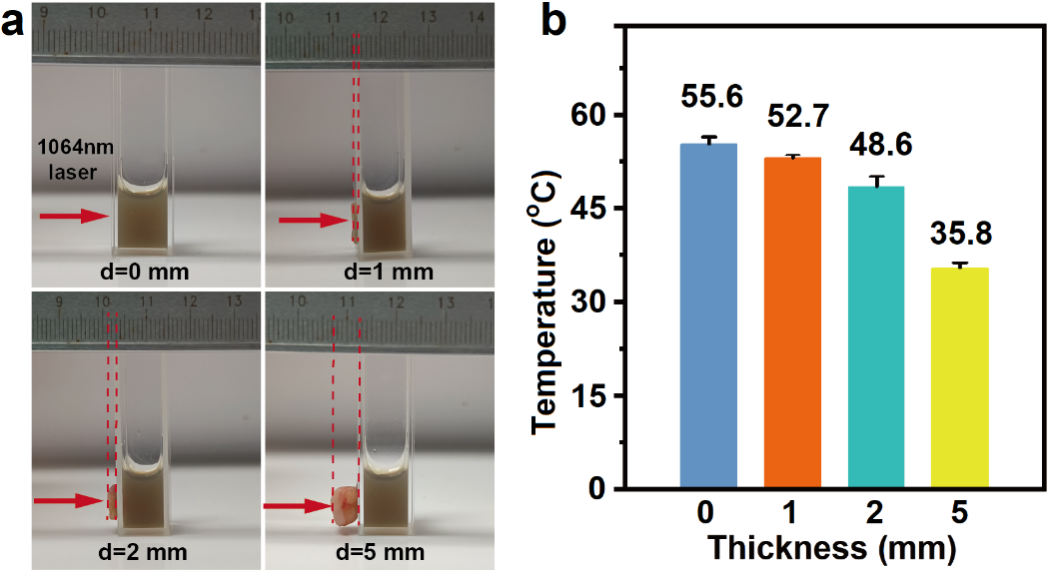


**Fig. S6** (a) Digital images of different tumor thicknesses (0, 1, 2, and 5 mm). (b) Temperature of FePS@PPF after the irradiation of 1064 nm laser (1.0 W/cm^2^) for 10 min.
